# The Effect of Rapeseed Oil Biopolyols and Cellulose Biofillers on Selected Properties of Viscoelastic Polyurethane Foams

**DOI:** 10.3390/ma17133357

**Published:** 2024-07-07

**Authors:** Tomasz Prociak, Dariusz Bogdal, Maria Kuranska, Olga Dlugosz, Mark Kubik

**Affiliations:** 1 Faculty of Chemical Engineering and Technology, Cracow University of Technology, Warszawska 24, 31-155 Cracow, Poland; maria.kuranska@pk.edu.pl (M.K.); olga.dlugosz@pk.edu.pl (O.D.); 2 Stanmark, Halki 6/1, 30-228 Cracow, Poland

**Keywords:** polyurethanes, viscoelastic foams, biopolyols, biofillers, foaming, physical-mechanical properties, acoustics

## Abstract

This paper presents the results of research on polyurethane viscoelastic foams (PUVFs) modified with biomaterials. This investigation looked at the effect of the biomaterials on the foaming processes, as well as the acoustical and selected physical-mechanical properties of the foams. Various types of rapeseed oil biopolyols and microcellulose were used to modify the materials. The analysis of properties covered a reference biopolyol-free sample and materials containing 10 wt.%, 20 wt.%, and 30 wt.% of different types of biopolyols in the mixture of polyol components. The biopolyols differed in terms of functionality and hydroxyl value (OH_v_). Next, a selected formulation was modified with various microcellulose biofillers in the amount of 0.5–2 wt.%. The PUVFs, with apparent densities of more than 210 kg/m^3^ and open-cell structures (more than 85% of open cells), showed a slow recovery to their original shape after deformation when the pressure force was removed. They were also characterized by a tensile strength in the range of 156–264 kPa, elongation at break of 310–510%, hardness of 8.1–23.1 kPa, and a high comfort factor of 3.1–7.1. The introduction of biopolyols into the polyurethane system resulted in changes in sound intensity levels of up to 31.45%, while the addition of fillers resulted in changes in sound intensity levels of up to 13.81%.

## 1. Introduction

Plastics, having advantageous properties as compared to other similar materials, are often used in various areas of life. In 2022, the global production of plastics was estimated to exceed 400 million tons [1], and the polymer market was worth more than USD 700 billion [2].

Polyurethane foams are among the most important foamed polymer materials. The low density and thermal conductivity of foamed polyurethanes, as well as their mechanical properties, make them excellent thermal and acoustic insulators [3,4]. One of the groups of polyurethane foams is viscoelastic foams (PUVFs), which are materials with a high comfort factor. They are characterized by a slow return to their original shape, which occurs as a result of deforming force removal, as well as resilience that generally does not exceed 20%. Their viscoelastic properties are determined by the values of glass transition temperatures, Tg, in particular, the temperature corresponding to the transition from the viscoelastic state into the elastic state [5].

The production of polyurethane foams is still strongly dependent on crude oil, and as a result, the polymer industry must adapt to increasingly restrictive regulations related to human and environmental concerns. In this context, the raw materials and technologies used to date are changing in favor of renewable raw materials and new processing technologies [6]. 

Searching for new developments has led, e.g., to proposals to replace petrochemical polyols with biopolys derived from vegetable oils. A wide range of vegetable oil derivatives are used for the modification of polyurethane (PUR) materials. The most important oils are highly unsaturated ones, mainly soybean, rapeseed, and sunflower oils, as well as castor and palm oils. Rapeseed oil is the most common in Europe. Therefore, its easy availability influences research work related to the use of hydroxyl derivatives of this oil for the modification of polyurethanes. Biopolyols are characterized by biodegradability and can be an equivalent substitute for traditionally used raw materials [7]. The structures of crude vegetable oils mostly do not contain functional groups that would be able to react with isocyanate to form a polyurethane bond. Therefore, it is necessary to functionalize such raw materials, which can be carried out, for example, by epoxidation [8], transesterification [9], transamidation [10], and other methods [11,12,13]. Such a modification results in the formation of hydroxyl groups, which can react with isocyanate groups. In contrast, an oil that does not require modification because it already contains hydroxyl groups in the chain structure is castor oil [14].

In view of the growing demand for newer types of polyurethane materials, it is especially important to improve their physicochemical and mechanical properties. For this purpose, various types of fillers are used to improve their properties by interacting with the polymer matrix. Nowadays, the most widely used fillers include carbon fibers [15], carbon nanotubes [16], poly(ethylene terephthalate)—(PET) [17], and silica [18]. It should be noted that the commonly used industrial fillers have specific properties that make it impossible to classify them as completely friendly to living organisms and the environment. In particular, it has been confirmed that once nanoparticles and carbon fibers enter the environment, they can easily penetrate the tissues and organs of living organisms. As a result of their accumulation, these organisms are exposed to health problems such as reduced reproduction or respiratory problems [19,20]. 

Another major problem associated with the use of the above-mentioned fillers is their low level of biodegradability, as well as their energetic and expensive life cycle [21]. The production of graphene nanoparticles, for example, is based on processes carried out using ultrasound or based on chemical reduction reactions. An analysis of the energy demand over the entire life cycle of carbon nanofibers compared with an equal weight of traditional materials, such as aluminum, steel, and polypropylene, has also been analyzed. It was found that the life cycle energy demand of carbon nanofibers is 13 to 50 times higher than that of, for example, equal-weight aluminum [22].

Life cycle analysis has confirmed that PET causes serious damage to human health and ecosystem quality [23]. In particular, PET microparticles released through degradation become a significant environmental problem. These particles have been detected in oceans, rivers, wastewater sediments, soil, and even table salt. High levels of exposure of marine organisms and humans to PET microparticles have been documented, but information on mammalian toxicity is still limited. The distribution and mode of specific accumulation of microplastic in tissues have been studied using fluorescent particles of polystyrene microplastic. Exposure of mice to particles with diameters of 5 µm and 20 µm has shown that microplastics accumulate in the liver, kidneys, and intestines, and the kinetics of accumulation in tissues and distribution pattern strongly depend on particle size. In addition, biochemical studies have confirmed that exposure to microparticles disrupts energy and lipid metabolism and also causes oxidative stress [24]. 

Even though commonly used fillers improve the physicochemical and mechanical properties of polyurethane foams, concern for human health, the environment, and sustainability justifies an increased demand for using more environmentally friendly fillers, such as cellulose [25]. 

This paper presents the possibility of obtaining durable viscoelastic polyurethane foams (PUVFs) with beneficial mechanical and acoustic parameters based on biopolyols and natural biofillers. Biopolyols made from rapeseed oil were used to obtain polyurethane materials. Three types of microcellulose were used as fillers. Microcellulose can be made from lignocellulosic raw materials such as wood, rice, straw, and hulls of various seeds [26]. Moreover, microcellulose is seen as a green material with industrial applications [27,28]. 

## 2. Materials and Methods

Open-cell viscoelastic polyurethane foams (PUVFs) were obtained by a one-step method. Two series of polyurethane foams differing in formulation were prepared. The first series of materials comprised products prepared using several types of petrochemical polyols, a catalyst, a surfactant, a molecular chain extender, a blowing agent, and three types of biopolyols, the content of which was set at three different levels (Table 1). This composition was a polyol premix. The biopolyols (BP DEG 112, BP DEG 188, BP DEG 256) used in the PUVFs were characterized by hydroxyl values (OH_v_) of 112, 188, and 256 mgKOH/g, respectively. The oxirane ring-opening agent was diethylene glycol. The symbols of the obtained biopolyols make reference to their OH_v_. The biopolyols were synthesized in the Department of Chemistry and Technology of Polymers of the Faculty of Chemical Engineering and Technology of the Cracow University of Technology [29,30,31].

The second series of materials was prepared using the same PUR system as in series 1 with a biopolyol (BP DEG 112) content of 20% in the polyol premix and three types of microcellulose fillers (ARBOCEL^®^ P4000X, ARBOCEL^®^ UFC100, ARBOCEL^®^ UFCM8) supplied by Rettenmaier Polska Sp. z o.o. (Warszawa, Poland) The contents of the fillers were set at four levels (Table 1). The reference material (REF) in series 1 was PUVF containing neither a biopolyol nor a filler. The average OH_v_ of the polyol premix to obtain the REF material was 225 mgKOH/g, and the average functionality was 4.21. The reference material in series 2 was a foam with biopolyol BP DEG 112, whose content was 20%, without a filler. The isocyanate component in all materials was polymeric diphenylmethane diisocyanate (PMDI). This research was carried out within the framework of an implementation doctorate involving collaboration between the scientific unit, Cracow University of Technology, and the industrial company Stanmark. The type and specific properties of the components used are the Stanmark company’s strict knowledge and cannot be disclosed at any stage of the presentation of the test results.

**Figure 1:**
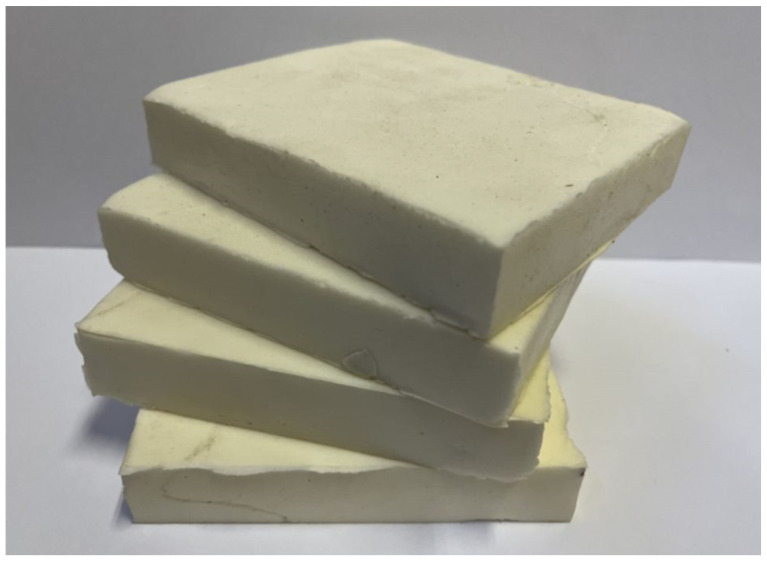
An overview image of PUVFs with a thickness of 20 mm.

To obtain a homogeneous mixture, all components were mixed for about 2 min at 5000 rpm. The prepared polyol premix, together with the polymeric diphenylmethane diisocyanate (PMDI), were mixed using a mechanical stirrer for a period of 4 s at a speed of 5000 rpm. Then, the reaction mixture was poured into a sealed aluminum mold, which was then placed in an oven and heated at 60 °C for 25 min. The mold was lined with a layer of polypropylene for easier extraction of the sample. Carbon dioxide (CO_2_) formed by the reaction of water with isocyanate was used as a foaming agent. Two versions of materials were prepared, approximately 10 mm and 20 mm thick. Materials with a thickness of 10 mm were cut into samples and used in mechanical tensile tests, whereas materials with a thickness of 20 mm (Figure 1) were cut into samples and used in both compression tests and acoustic tests. The apparent density of all PUVFs was 210–220 kg/m^3^.

To verify the results and evaluate the influence of environmental conditions on the foaming process, further analyses of polyurethane materials were performed. Cream time, rise time, gel time, and tack-free time were determined. The cream time was measured using a timer, determining the time from the moment the raw materials were mixed until the mixture reached a creamy state. The gel time was measured from the moment the raw materials were mixed until it was possible to pull the “threads” out of the reaction mixture with a baguette. The rise time was determined from the moment the raw materials were mixed until the foam reached its maximum volume. The tack-free time was measured from the moment the raw materials were mixed until the foam surface was dust-dry. 

The properties of the resulting polyurethane foams were evaluated by measuring their return time, following the IKEA standard. In this test, the foam is compressed to 75% of its original height and held in that state for 60 s. Once the load is removed, the time it takes for the foam to recover to 90% of its initial height is recorded. 

An analysis of the percentage of closed cells in the obtained materials was also carried out. For this purpose, the pycnometer method and the ISO 4590:2016 [32] procedure were used.

A scanning electron microscope (SEM) TM3000 (Hitachi, Tokyo, Japan) helped to analyze the cellular structures of the PUVFs.

The chemical structures of the materials were analyzed based on absorbance spectra using a Nicolet iS5 Fourier transform infrared (FTIR) spectrophotometer (Thermo Fisher Scientific, Waltham, MA, USA). Each sample was scanned eight times in the 4000–400 cm^−^^1^ wavelength range 10 days after the material was pulled out of the mold. 

**Figure 2:**
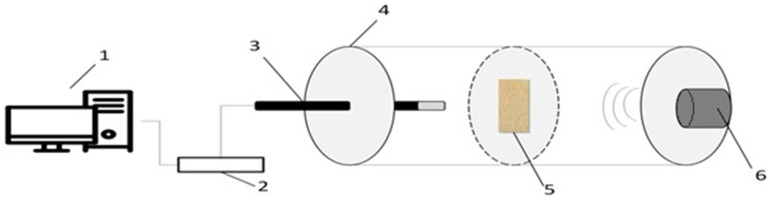
Schematic diagram showing how the sound properties of PUVFs were studied (1—computer with audio software Audacity 3.1.3, 2—interface, sound-level preamplifier Focusrite Scarlett 4i4 3rd generation, 3—microphone (output device, Precision Reference Microphone, PreSonus, Frequency Response from 20 Hz to 20 kHz), 4—soundproofed measurement tunnel (Kundt’s tube), 5—acoustic barrier with sample location, 6—loudspeaker Sony SRS-XB12.

The acoustic analysis examined the sound insulation properties of the materials. Figure 2 illustrates how the acoustic tests of PUVFs were performed.

The percentage changes in sound intensity level (after passing through the PUVF) between the reference sample and the modified samples were compared (Equation (1)).

(1) $$R_{s}=\frac{L_{0}-L_{s}}{L_{0}}100\;[\%]$$

For series 1, the following parameters were defined:

*R_s_*—percentage change in sound intensity level [%];

*L*_0_—sound intensity level for the REF sample [dB]; 

*L_s_*—sound intensity level for the samples modified with biopolyols [dB].

For series 2, the following parameters were defined:

*R_s_*—percentage change in sound intensity level [%];

*L*_0_—sound intensity level for the BP1/20 sample [dB];

*L_s_*—sound intensity level for the samples modified with microcellulose fillers [dB].

In order to perform the measurement, a 60 mm diameter impedance tube and rigid tube/wall duct were used with a loudspeaker (input device, Sony SRS-XB12, Tokyo, Japan) at one end and a microphone (output device, Precision Reference Microphone, PreSonus, Frequency Response from 20 Hz to 20 kHz, Baton Rouge, LA, USA) at the other end of the tube. An acoustic baffle made from 20 mm thick expanded polystyrene with a hole for the sample to be analyzed was placed inside the tube. The tested samples were cuboids with the following dimensions (W—width, T—thickness, L—length): approximately 25 (W) × 20 (T) × 50 (L) mm. The distance between the loudspeaker and the sample was 25 cm and was the same as the distance between the sample and the microphone. A probe microphone was used to measure the maxima and minima of the standing wave sound pressure in the tube at a single frequency and was connected to a Focusrite Scarlett 4i4 3rd generation microphone preamplifier (High Wycombe, UK). Measurements with Audio Inter Sound were taken at 63 Hz, 125 Hz, 250 Hz, 500 Hz, 1000 Hz, 2000 Hz, 4000 Hz, and 8000 Hz.

An analysis of mechanical properties, including tensile and compressive strength, was also made. Tensile tests were conducted according to EN ISO 1798:2008 [33] for samples of approximately 10 (W) × 10 (T) × 95 (L) mm, with a tensile speed of 200 mm/min and a handle distance in the starting position of 50 mm. Mechanical compression tests were performed according to PN-EN ISO 3386-1:2000 [34] for samples of approximately 25 (W) × 20 (T) × 50 (L) mm. Both tests were carried out with a Z005 TH Allround-Line testing machine (Zwick Roell, Ulm, Germany). Each sample of the test material was compressed three times at 75% deformation before the proper measurement. The time between measurements was 5 min. In that way, the foam was given enough time to return to its original dimensions. Based on the results of the fourth compression, the hardness of the foam (the compression stress of the samples at 40% deformation) and the comfort factor of the foam (stress at 65% compression of the sample/stress at 25% compression of the sample) were found. The comfort factor is one of the indicators of the material’s comfort. The higher the factor, the softer the material at low compression, combined with higher hardness at high compression.

## 3. Results and Discussion

Twenty-two PUVFs were obtained that differed in their compositions. Those were one reference foam without biopolyol or filler, nine foams with three biopolyols at three different share levels, and 12 foams with three fillers at four different share levels. All the products were cream-colored with varying intensities depending on the composition.

**Figure 3:**
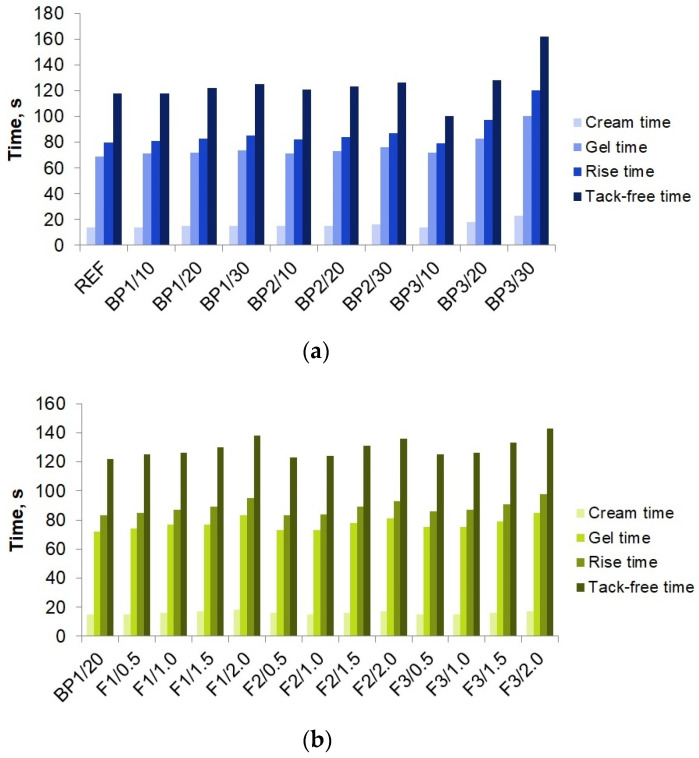
Characteristic times for foaming processes of foam series 1 (**a**) and series 2 (**b**).

Figure 3 shows the results of the analysis of cream, gel, rise, and tack-free times measured during the processes of obtaining PUVFs in two batches. 

Enriching the formulation with the biopolyols resulted in an extension of the cream, gel, and rise time. However, in the case of BP1 and BP2, the extensions of all times were by less than 8 s. The analysis of the tack-free time showed that the addition of the biopolyols resulted in an extension of the measured times in all cases except for the material with a 10% content of BP3 (BP3/10). It was observed that the times became longer as the contents of the biopolyols in the mixture increased. The difference between the tack-free time of BP3/10 and BP3/30 was 62 s. The overall lengthening of the above-mentioned times was most likely due to the high viscosity of the biopolyols used, which explains a greater time extension at a higher biopolyol content.

The addition of fillers to the formulations in all variants (different types and amounts of filler) resulted in a lengthening of the gel, rise, and tack-free times compared with the reference material. The cream time increased as the filler content grew, but only in the case of P4000X. An increase in the contents of the other fillers did not result in a significant extension of the cream time. The gel, rise, and tack-free times increased with the increasing content of each filler. This is due to a decreasing reactivity of the mixture caused, as in the case of the biopolyols, by an increasing viscosity of the polyol premix. The longest times were found for the materials obtained with UFCM8, the content of which was 2% (F3/2.0). This sample was tack-free after 143 s.

Bartczak et al. [35] also studied the effect of fillers (coffee grounds and sawdust) on the foaming processes of rigid polyurethane foams. An addition of a filler in the range of 2.5 to 10% by weight changed the time the system needed to reach the gelation stage. As the filler content increased, the gel time increased, too, with the most significant changes observed for samples containing the highest filler concentration. An introduction of sawdust, starting at 5% by weight, delayed the foam rise termination. The amount of the filler used was a key factor in determining rise time. The longer gelation and foam rise termination times were likely due to reduced pore expansion during foaming as a result of the increased viscosity of the blend (polyol blend with sawdust added) and the formation of additional nucleation centers, promoting increased bubble formation during the reaction. 

Similar observations were made by Leszczynska et al. [36]. The authors found that an addition of natural filler particles to a PUR polyol premix caused a significant change in its viscosity. This change was additionally influenced by factors such as the size, shape, and surface area of the filler particles. 

**Figure 4:**
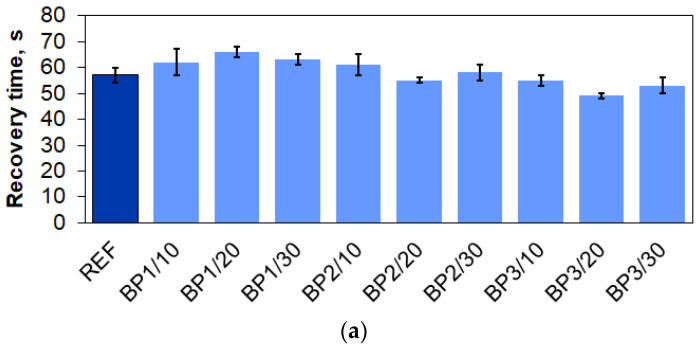

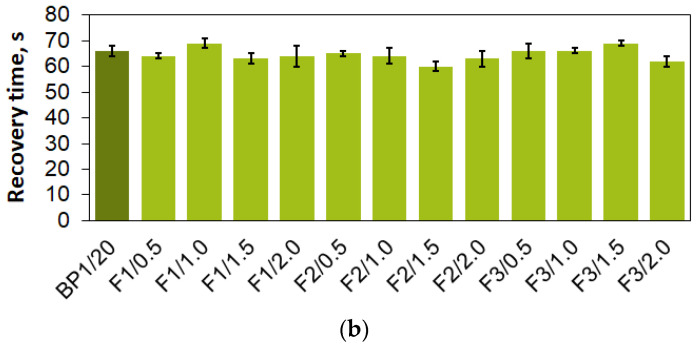
Recovery time of PUR materials from series 1 (**a**) and series 2 (**b**).

The recovery time values of the obtained polyurethane materials were measured (Figure 4). In series 1, the recovery times of the PUVFs with BP1 extended slightly more than in the case of the reference material. When BP2 was used, the times were similar to the reference, and the use of BP3 decreased them slightly compared with the reference material. The decreased recovery time is most likely related to better cross-linking of the material. In series 2, there was no significant effect of individual fillers on the recovery time compared with the reference. The results confirm the viscoelastic nature of the foams [37]. 

**Figure 5:**
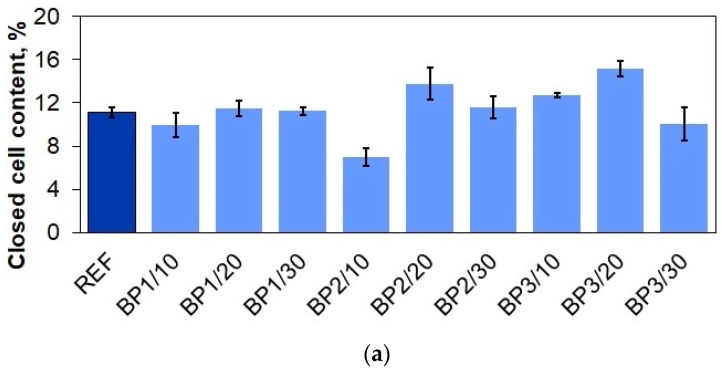

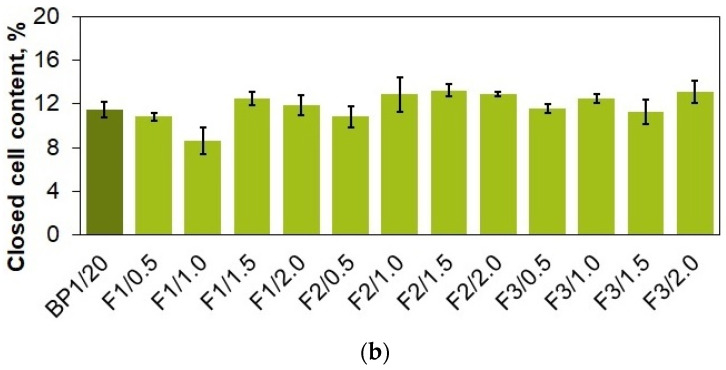
Closed cell contents in PUR materials from series 1 (**a**) and series 2 (**b**).

In our experiment, an addition of each biopolyol slightly changed the percentage content of closed cells (Figure 5a). Despite this, all materials had low contents of closed cells (7.0–15.2%). The largest changes were observed in the BP2/10 and BP3/20 samples. The first sample experienced a reduction in the number of closed cells from 11.1% to 7.0%, while in the material, with the addition of the second biopolyol, the number of closed cells increased from 11.1% to 15.2%. These changes were minimal and had little effect on the acoustic or mechanical properties. As a result, all the materials preserved the desired hardness, comfort factor, and viscoelastic properties. Similar results were obtained by Hejna et al., who used a biopolyol synthesized from crude glycerol and castor oil to obtain polyurethane foams. The closed cell content of the reference foam was 94%, while replacing 52.5 wt.% of the petrochemical polyol with the biopolyol increased this value to >95%. The 70% biopolyol content, on the other hand, reduced the number of closed cells to 85% [38]. 

In the case of the cellulose-modified PUVFs, the changes in the number of closed cells were even smaller (+/−1%). Only in the case of sample F1/1.0 was there a noticeable change in the number of closed cells from 11.5% to 8.7% (Figure 5b). Javni et al. tested fillers in the form of nanosilica, microsilica, nanoclay, and microclay and did not observe major changes in the number of encapsulated cells either. All materials tested, regardless of the type and amount of filler, had <5% of closed cells. On the other hand, based on low airflow, they found that not all cells were fully open. Two foams with nanoclay were irregular and had distorted cell shapes, which was probably related to viscosity and polymer-filler interactions [39].

**Figure 6:**
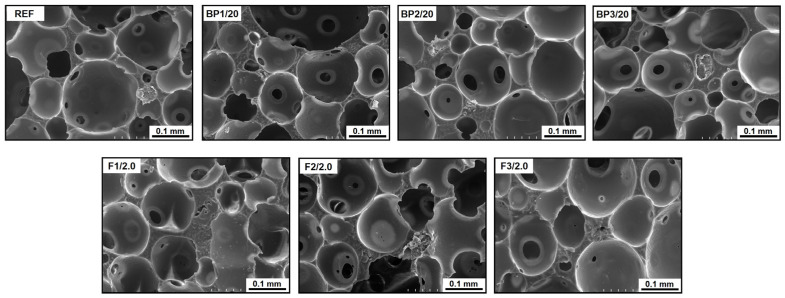
SEM images of PUVFs (REF, modified with biopolyols and both biopolyol and biofillers).

The open-cell structures of the materials obtained in the present work are perfectly illustrated by the scanning electron microscope (SEM) images shown in Figure 6.

**Figure 7:**
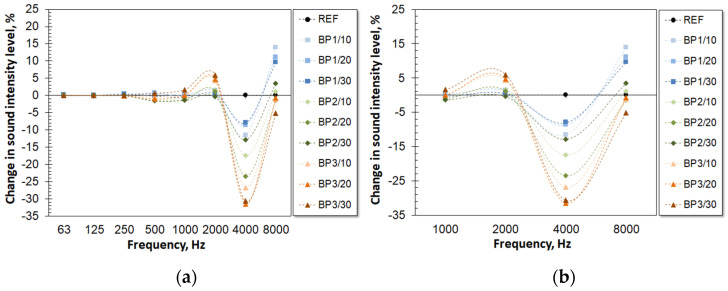

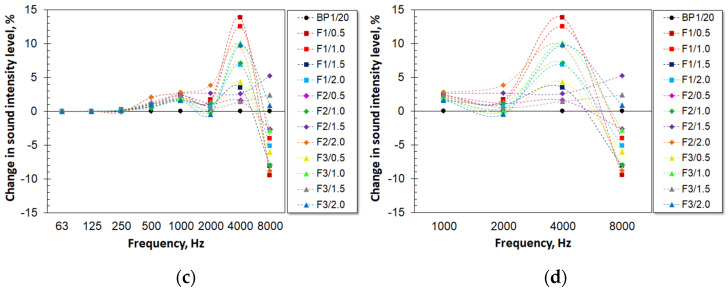
Change in sound intensity level of PUVFs for the frequencies 63 Hz, 125 Hz, 250 Hz, 500 Hz, 1000 Hz, 2000 Hz, 4000 Hz, 8000 Hz: series 1 (**a**), Detail series 1 (**b**), series 2 (**c**), Detail series 2 (**d**).

In the next stage, the acoustic properties of the foams were studied by looking at the change in sound intensity level of the tested materials for the following frequencies: 63 Hz, 125 Hz, 250 Hz, 500 Hz, 1000 Hz, 2000 Hz, 4000 Hz, and 8000 Hz (Figure 7). 

The positive values indicate an improvement in acoustic insulation properties compared with the reference material.

For series 1, the results for a frequency of 4000 Hz show that the PUVFs that had been prepared with biopolyols had slightly poorer sound intensity levels compared with the reference foam. This may be a result of the cellular structures of the PUVFs. The ability of a material to absorb sound at a certain frequency depends on the number of open cells and their sizes corresponding to the absorbed wavelength and geometry. Acoustic waves propagating in a material cause vibrations of the air inside cells and thin cell walls. Sound energy is reduced as a result of friction forces between the air stream and the cell wall, resulting in its conversion into thermal energy [40]. Theoretically, foam BP2/10, with its low content of closed cells, should offer better acoustic insulation. However, in this case, the sound intensity level was almost identical at most frequencies, except at 4000 Hz, where it was 17.55% higher compared with the REF sample. This means that such small changes in the number of closed cells do not affect the sound intensity level changes. However, it was observed that an increasing OH_v_ of the biopolyols led to a decrease in the acoustic insulation efficiency of the material at 4000 Hz. The most beneficial changes in sound intensity level were observed for BP1 materials at 8000 Hz, and they were 13.89% for BP1/10, 11.12% for BP1/20, and 9.64% for BP1/30, respectively. The most negative change in sound intensity level (31.45%) was observed for sample BP3/20. The materials with BP3 had better acoustic insulation in the 1000–2000 Hz frequency range and were poorer at 4000 Hz in comparison to the REF sample. Since smaller cells absorb short waves better and larger cells absorb long waves better, it can be concluded that the BP3-containing materials had an increased number of larger cells in comparison to the REF sample. In addition to the characteristic cellular structure, this effect may be enhanced by an increasing stiffness of the polyurethane matrix resulting from differences in the chemical compositions. The smallest changes in sound intensity level were for the samples BP1/20, BP1/30, and BP2/30, which indicates the most similar structure to the REF sample. Similar observations were made by Andersson et al., who investigated the effect of adding hyperbranched polymer (HBP) to a flexible polyurethane foam. By having an OH_v_ of 235–255 mgKOH/g, HBP forced an increase in the amount of isocyanate in the polyol premix to keep the NCO index. As a result, the mechanical properties were improved, and the hardness increased. In addition, as the amount of HBP increased, a greater acoustic reduction in the 500–1600 Hz range was observed. Since there were no apparent differences in the cellular structures, the authors speculated that the improved acoustic properties were due to the morphologies and chemical compositions of the foams [41]. 

In series 2, the most significant changes were also observed at 4000 Hz and 8000 Hz. However, unlike the foams with the biopolyols, these foams insulated acoustic waves more effectively than the BP1/20 foam, regardless of the filler type. The foam without fillers exhibited the worst acoustic properties at nearly all tested frequencies, except at 8000 Hz. The addition of the microcellulose fillers increased PUVFs’ reduction in sound intensity levels in the 500–4000 Hz range, to which the human ear is most sensitive. It could also be observed that the addition of fillers caused a change in the trend compared with biopolyols. In the case of fillers, a favorable change in sound level occurs at 4000 Hz and a negative change at 8000 Hz. The largest differences in sound intensity level change were observed for F1/0.5 at 13.81% for 4000 Hz and 9.52% for 8000 Hz, respectively. It confirms that both a filler type and a small difference in its content can considerably affect its acoustic properties but also its dispersion level. Hasani Baferani et al. noted a correlation between the degree of dispersion of carbon nanotubes (CNT) and improved acoustic properties. The scientists dispersed the filler in the sonication process. Increasing the time of the sonication resulted in an improved dispersion of the filler, which in turn led to improved acoustic energy absorption efficiency. According to the researchers, increasing the CNT sonication time caused an increase in the number of PU-CNT interfaces, which reduced the number of viscous movements of PU segments in the presence of a sound wave [42].

**Figure 8:**
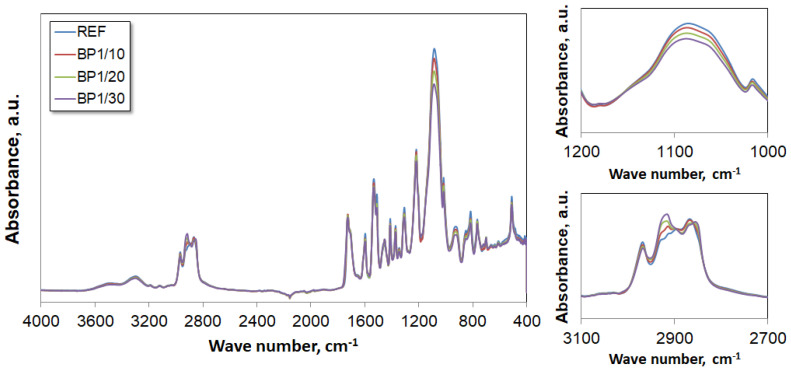
FTIR spectra of REF and biopolyol-modified PUVFs (BP1).

The Fourier transform infrared (FTIR) spectroscopy analysis confirmed the presence of groups characteristic of PUVFs in the chemical structures of the tested materials. The FTIR spectra are shown in Figure 8. Characteristic peaks are visible, confirming that the desired products were obtained. Peaks appearing at 1075 cm^−1^ confirm the presence of stretching vibrations in the C-O-C group. The peaks located at 1217 cm^−1^ confirm the presence of stretching vibrations of C(O)O-C groups. The peaks located at 1412 cm^−1^ correspond to deformation vibrations of C-H groups. Stretching and bending vibrations of N-H groups present in urethane bonds are confirmed by a strong band at 1530 cm^−1^ and stretching bands at 3292 cm^−1^. The band at 1595 cm^−1^ corresponds to C=C bond vibrations of aromatic isocyanate groups. Stretching vibrations of the C=O carbonyl group of the urethane bond appear at 1704 and 1722 cm^−1^. In the spectra, there is no peak in the 2270–2240 cm^−1^ band of NCO groups, indicating that all NCO groups reacted. The presence of bands between 2889 and 2912 cm^−1^ is due to both symmetric and asymmetric stretching vibrations of aliphatic -CH_2_- groups from the used polyols [43,44,45,46,47].

**Figure 9:**
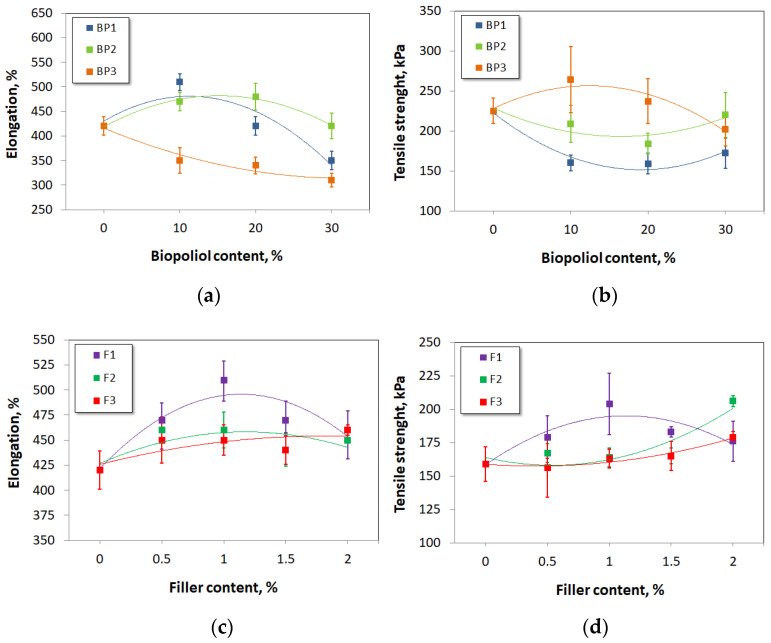
Elongation at break and tensile strength of reference material and materials modified with biopolyols (**a**,**b**) and biofillers (**c**,**d**).

The mechanical properties (elongation at break and tensile strength) of the obtained foams are shown in Figure 9. For the REF foam, the elongation was 420%. When biopolyols BP1 and BP2 were used, the elongation initially increased and then decreased to 350% and 420%, respectively, for the biopolyol content of 30%. For BP1, the low OH_v_ and functionality of 1.74 led to the formation of short cross-linked polymer chains, revealing an increased elasticity of the material at the cost of a reduced tensile strength. In addition, the biopolyol had so-called hanging chains in its structure, which may result in a plasticizing effect of the polyurethane matrix [48]. However, increasing the amount of the biopolyol progressively resulted in a decreasing elongation. A better elongation of petrochemical polyols could be due to their higher molecular weight, i.e., longer chains. In the case of BP2, its properties were most similar to the properties of the polyol premix the REF material was made from, and the changes in values were the lowest. Also, in the case of tensile strength for the BP1 and BP2 biopolyols, the relationship of this parameter to their content had the same course. Initially, when their contents were 0%, the tensile strength was 225 kPa. When their contents increased, the tensile strength initially decreased, but at the highest biopolyol amount (30%), this parameter increased slightly. When the BP3 biopolyol was used, the elongation decreased to 310%, and the tensile strength initially increased. After reaching 264 kPa at 10% BP3 content, the tensile strength decreased to 202 kPa. Functionality at 5.68 and OH_v_ of 256 mgKOH/g resulted in the formation of more branched chains. That made the material more durable but less flexible, explaining a noticeable correlation between elongation at break and tensile strength (Figure 9a,b).

In the case of series 2, it was observed that when fillers F1 and F2 were used, elongation increased from an initial value of 420% (for the sample without a filler), reaching a maximum at a filler content of 1%, and then decreased. The highest elongation was found for filler F1: 510%. When filler F3 was used, no established relationship between elongation and filler content was observed, but in each case, elongation was greater than in the reference material. 

In the absence of fillers, the tensile strength was 159 kPa. When the F1 filler content was increased, the inflection point was observed at 1%, and the tensile strength reached a record value of 204 kPa. At this point, the parameter decreased as the F1 filler content increased. The dependence of the tensile strength on the F1 content resembles the dependence of the elongation on the F1 content. Similarly, the tensile strength increased with increasing contents of the fillers F2 and F3. Even though the use of filler F2 led to a material with a tensile strength of 206 kPa, this material had inferior elongation properties. Husainie et al. used cellulose, chitin, hazelnuts, and eggshells as natural fillers for rigid polyurethane foams. These fibers were added at 1%, 2.5%, and 5% by weight. The elongation remained at the level of the control formulation for 1% of the fillers, and it decreased with their increasing amounts. At the same time, the fillers had a positive effect on the strength of the foam. That effect was caused by three phenomena. Adding a filler to the foam helped increase the resistive surface area. Moreover, it helped reduce the stretching mobility of the polymer chains. At low amounts, the filler could effectively interact with the PUR matrix, creating high matrix-filler reinforcements. Excess filler, unfortunately, increased the number of empty spaces in the foam, which prevented the formation of effective matrix–filler interactions, resulting in a decrease in tensile strength [49]. 

The results on the elongation at break and tensile strength of the materials without and with fillers are shown in Figure 9c,d.

**Figure 10:**
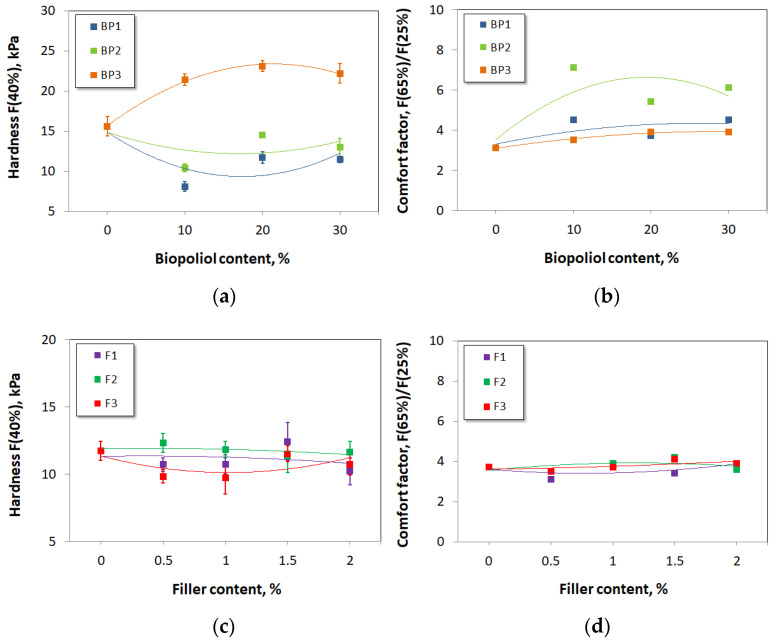
Hardness and comfort factor of reference material and materials modified with biopolyols (**a**,**b**) and biofillers (**c**,**d**).

It was found that the hardness was 15.6 kPa in the absence of the biopolyols. The influence of the contents of BP1 and BP2 was not clear. The hardness of the materials was significantly lower than in the case of the REF material and the material with BP3. This could be related to several factors, e.g., the content of closed cells, the presence of so-called hanging chains in the structure of the biopolyol, and the fatty acid chain in its role as a soft segment performing plasticizing functions. Polyurethane materials that have been synthesized from polyester polyols are usually characterized by lower hardness than those made from commercial polyether polyols [50]. As the BP3 content increased, the hardness values also increased until they reached 23.1 kPa at a filler content of 20%. This is due to the high functionality and hydroxyl value, which result in the formation of more branched chains. In addition, in order to keep the isocyanate index constant, more PMDI was used, which increased the number of rigid segments in the materials [51]. The hardness decreased slightly when the content of BP3 exceeded 20%. The lowest value of hardness was achieved for BP1 at 10% (8.1 kPa), as shown in Figure 10a. When no biopolyol was used, the comfort factor was 3.1. The highest comfort factor value was achieved with biopolyol BP2 at its content of 10%. However, the effect of BP2 on comfort factor values was unclear. When the contents of BP1 and BP3 increased, the comfort factor also increased slightly to 4.5 and 3.9, respectively. All foams met the requirements, and their comfort factor values were >2.5 (Figure 10b). Zlatanic et al. also noted that the comfort factor increased with an increasing soybean oil biopolyol content [52]. 

The results of the hardness tests with and without fillers are shown in Figure 10c. As the content of filler F3 increased from 0 to 1%, the hardness dropped from 11.7 to a minimum of 9.7 kPa. Then, it increased slightly but remained at a similar level, which is a great advantage of the cellulose fillers used. Figure 10d presents that the influence of the filler content on the comfort factor was negligible. The same comfort factor value was seen for the reference material without a filler addition.

## 4. Conclusions

Rapeseed oil biopolyols and cellulose biofillers were successfully used to obtain viscoelastic polyurethane foams (PUVFs) with beneficial mechanical and acoustical parameters.

The addition of the biopolyols increased cream, gel, rise, and tack-free times as a result of increased viscosity. The fillers, regardless of their type and quantity, extended the foaming time. The highest increase was achieved with a 2% addition of UFCM8 microcellulose biofiller. Small changes in the closed-cell content were observed with each addition of the biopolyols and biofillers. However, the impact of closed-cell content on the acoustic and mechanical properties of the foams was minimal, and their properties were preserved.

An introduction of the biopolyols to the polyurethane system slightly affected changes in the sound intensity levels after passing through the PUVF samples for frequencies of 2000 Hz and below. In general, foams with fillers showed better acoustic insulation at most of the frequencies tested, especially in the range of 500–4000 Hz, to which the human ear is most sensitive. In both cases, the greatest changes were observed at 4000 Hz and 8000 Hz.

The addition of the biopolyols had a variable impact on the mechanical properties of the PUVFs, as indicated by OH_v_ and functionality. The presence of so-called “hanging chains” in the structures of the biopolyols and a fatty acid chain as a soft segment with plasticizing functions also influenced the mechanical properties of the PUVFs. In some cases, the addition of the biopolyols significantly improved such properties as elongation at break (510% for BP1/10), tensile strength (264 kPa for BP3/10), hardness (8.1 kPa for BP1/10), and comfort factor (7.1 for BP2/10). In the case of elongation at break and tensile strength, all the fillers led to an improvement in these parameters. The result was particularly good for the F1/1.0 material, where elongation at break was 510% and tensile strength was 204 kPa. The effect of the fillers on the hardness and comfort factor of the foams was marginal. 

A replacement of petrochemical polyols with rapeseed oil biopolyols can offer not only environmental benefits but also stable viscoelastic polyurethane materials with similar or more preferable properties. Such materials can be further improved by adding biofillers.

## Figures and Tables

**Table 1:** Compositions of polyol premixes in two series of PUVF.

SERIES 1				
Type of Biopolyol	Hydroxyl Value (OH_v_), mgKOH/g	Functionality	Percentage Participation in the Polyol Premix, %	Symbol of Sample
BP DEG 112	112	1.73	10	BP1/10
			20	BP1/20
			30	BP1/30
BP DEG 188	188	4.35	10	BP2/10
			20	BP2/20
			30	BP2/30
BP DEG 256	256	5.68	10	BP3/10
			20	BP3/20
			30	BP3/30
**SERIES 2**				
**Type of Filler**	**Percentage Participation in the Polyol Premix, %**			**Symbol of Sample**
Microcelullose P4000X	0.5			F1/0.5
	1.0			F1/1.0
	1.5			F1/1.5
	2.0			F1/2.0
Microcelullose UFC100	0.5			F2/0.5
	1.0			F2/1.0
	1.5			F2/1.5
	2.0			F2/2.0
Microcelullose UFCM8	0.5			F3/0.5
	1.0			F3/1.0
	1.5			F3/1.5
	2.0			F3/2.0

## Data Availability

The original contributions presented in the study are included in the article, further inquiries can be directed to the corresponding authors.
